# Integrating knowledge-based planning and noncoplanar oblique VMAT arcs: A study of dose to the heart and immune cells in thoracic radiotherapy^☆^

**DOI:** 10.1016/j.tipsro.2025.100301

**Published:** 2025-01-05

**Authors:** Brennan Diedrich, Justin Roper, Benjamin Hopkins, Sibo Tian, Shadab Momin, Eduard Schreibmann, Aparna H. Kesarwala, Kirk Luca

**Affiliations:** Department of Radiation Oncology, Emory University, Atlanta, GA, USA

**Keywords:** Non-Small Cell Lung Cancer, Effective Dose to Immune Cells, Knowledge-Based Planning, RapidPlan, Noncoplanar

## Abstract

**Background:**

Recent patient studies have linked higher immune cell doses with worse quality of life and survival. For thoracic radiotherapy, heart dose is a major contributor to the effective dose to immune cells (EDIC).

**Purpose:**

This study investigates heart and immune cell doses for plans optimized using a cardiac-sparing knowledge-based planning (KBP) model and the impact of carefully crafted beam geometry.

**Methods:**

Sixteen stage III NSCLC patients previously treated to 60 Gy in 30 fractions using coplanar VMAT arcs were replanned using a cardiac-sparing KBP model with either the clinical field arrangement or noncoplanar oblique arcs that prioritize heart sparing. The cardiac-sparing KBP model consisted of fifteen substructures, all of which were used during optimization. All plans were normalized to 95% PTV coverage at 60 Gy. Statistical significance was assessed for EDIC (Jin Model), along with mean doses to the heart, lungs, body, and both the mean dose and D0.03 cc values for cardiac substructures, using the Wilcoxon signed-rank test.

**Results:**

Compared to the clinically treated plans with the same beam geometry, cardiac-sparing KBP reduced mean heart dose from 8.50 Gy to 4.09 Gy and EDIC from 4.27 Gy to 3.81 Gy (p < 0.001). For the novel arcs, the mean heart dose was reduced to 3.70 Gy, significantly lower than KBP with clinical beam geometry (p = 0.001). EDIC, however, was equivalent. No statistically meaningful differences were observed for the remaining organs at risk, and all plans met institutional planning goals.

**Conclusion:**

Cardiac-sparing RapidPlan is a valuable tool for reducing heart dose and lowering EDIC in NSCLC patients. Additional heart sparing is possible by strategically crafting noncoplanar oblique beams to minimize heart dose.

## Highlights

• Tailored knowledge-based planning models can enhance dosimetric outcomes.
• Beam geometry and optimization settings are crucial for radiation treatment planning.
• Choosing beam angles that position the heart at a deeper depth dose reduce EDIC.

## Introduction

Lung cancer is the leading cause of cancer-related fatalities in the United States [1], with non-small cell lung cancer (NSCLC) accounting for 80% of all cases [2]. Radiotherapy is indicated for more than 60% of patients with NSCLC at least once during their disease course [3]. Concurrent chemoradiation followed by immunotherapy is the standard of care in patients with locally advanced NSCLC [4], [5], [6]. Consequently, many strategies have been investigated to improve radiotherapy plan quality [7], [8]. Radiotherapy for lung cancer patients poses unique challenges due to the radiosensitivity of organs-at-risk (OAR) within the thoracic cavity, including the heart, healthy lung tissue, esophagus, and spinal cord [9]. Volumetric modulated arc therapy (VMAT) is a commonly used technique for definitive lung cancer radiotherapy [10], as it enables dose sculpting that can preferentially minimize exposure to the most radiosensitive organs and tissues that intensity modulated radiation therapy and three dimensional techniques do not offer [11]. While high dose conformity is often achieved with VMAT, there is variability in the spatial distribution of the lower isodose levels.

Beam geometry plays a critical role for shaping the isodose distribution in lung treatment plans. Half arcs are commonly used to minimize radiation exposure to the contralateral lung. This approach is generally collision-free and straightforward to implement, making it an efficient choice for treatment planning. However, this approach may not be optimal for all lung cases, particularly for tumors located anteriorly or posteriorly. In such instances, half arcs can result in unnecessarily long beam paths to the tumor, increasing the dose to critical OARs. Prior studies have demonstrated that noncoplanar beam geometry can reduce dose to OARs and enhance conformity, which are critical factors in lung treatment planning [12], [13]. Hamilton et al. investigated the effects of field geometry on posterior lung tumors and discovered that using noncoplanar oblique arcs significantly improved the dose conformity in lung stereotactic body radiation therapy, while also reducing doses to OARs, compared to lateral half-circular arcs. This study underscores the vital role of beam geometry for enhancing plan quality [14].

Optimization settings are critical to plan quality, as inadequate treatment planning strategies can result in suboptimal dose distributions due to conflicting objectives between the target structures and the OARs, potentially compromising tumor control and increasing the risk of normal organ toxicities [15]. Manually configuring optimization settings is both labor-intensive and complex, prompting many clinics to adopt automated planning solutions [16]. Knowledge-based planning (KBP) has gained popularity in the radiotherapy community by helping to automate high-quality treatment plans quickly and consistently [17], [18], [19]. There are commercially available KBP software programs which provide dose distribution estimates for each structure based on data-driven algorithms [20]. There are a few approaches to data-driven radiotherapy treatment planning: atlas-based, model-based, and deep learning-based methods [21], [22]. Each approach uses data from past treatment plans to establish correlations among various factors such as tumor location, size, patient anatomy, and prescribed dosage. The predictive models can then recommend optimization settings based on similar previous cases. For consistent results, KBP should be used only for plans with similar characteristics to those used in the training set [21]. However, consistency alone does not guarantee superior plan quality, as the performance of KBP models is dependent on the quality of the training dataset, which is influenced by beam geometry and optimization settings. This raises important questions about the use of published KBP models in clinical practice, particularly when combined with custom beam arrangements, as new clinical data may shift the priorities of treatment goals.

Plan evaluation for lung radiotherapy can be challenging due to the proximity of numerous nearby OARs. However, one often overlooked factor is the radiation dose to immune cells. Multiple studies in recent years have provided a foundation of understanding the prognostic impact of radiation to immune cells. A secondary analysis of Radiation Oncology Task Group (RTOG) 0617 by Jin et al. identified a correlation between immune cell dose and overall survival in patients with NSCLC [23]. The study defined a model to calculate the effective dose to immune cells (EDIC), which is an equivalent uniform dose to the entire blood based on radiation doses received by all blood-containing organs [23]. This model primarily focuses on radiation doses to key organs like the heart and lungs, while considering total body integral dose and the fractionation schema (Eq. (1)). From this equation, mean heart dose is shown to be one of the main factors that contribute to EDIC for thoracic radiotherapy patients. EDIC has been identified as a significant risk factor for overall survival [23]. The Jin model has been validated as a prognostic model, as well as by other studies that have produced similar EDIC models and reinforced the correlation with lymphoma [24], [25]. Higher radiation doses to cardiac structures have also been associated with reduced overall survival rates [26]. Building on a previous study that demonstrated the benefits of cardiac-sparing KBP to reduce heart dose [27], the aim of the present work is to further investigate cardiac-sparing KBP in conjunction with beam geometry. This study expands on prior research by exploring not only the reduction in heart dose but also the effects on EDIC, providing a more comprehensive evaluation of treatment planning strategies for improved patient outcomes.

(1) $$\text{EDIC}\;={\;\text{B}}_{\text{1}}\%\times \;\text{MLD}\;+{\;\text{B}}_{\text{2}}\%\times \;\text{MHD}\;+\left[{\text{B}}_{\text{3}}\%+{\;\text{B}}_{\text{4}}\%\times {\;\text{k}}_{\text{1}}\times {\left(\text{n}/\text{45}\right)}^{1/2}\right]\times \;\text{ITDV}\;/\left(\text{61}.\text{8}\;\times \;\text{1}0^{\text{3}}\right)$$

Equation (1). B represents the four main blood-containing organs (lung, heart, great vessels, and vessels/capillaries in all other organs). B_1_% = 0.12, B_2_% = 0.08, B_3_% = 0.45, B_4_% = 0.35. Dose effectiveness factor for small vessels and total body volume estimation is represented by k_1_ = 0.85 and n is the number of fractions. MLD = Mean Lung Dose, MDH = Mean Heart Dose, ITDV = Integral Total Dose Volume.

## Methods

### Case selection

This retrospective study included a cohort of 16 clinical cases previously treated with conventional VMAT radiotherapy for stage III non-small cell lung cancer (NSCLC) from 2013 to 2021. These 16 cases were part of a previous research study conducted at our institution that investigated a cardiac-sparing RapidPlan – Eclipse^TM^ treatment planning systems knowledge-based planning software – model to enhance the dosimetric sparing of cardiac substructures [27]. RapidPlan is a knowledge-based treatment planning system that utilizes the previously approved treatment plans to build a dose-prediction model for each structure. Each case was prescribed a dose of 60 Gy in 30 fractions. Approval for this study was obtained from the Institutional Review Board under protocol #00006087.

### Model Creation

The cardiac-sparing RapidPlan model utilized in this study is described in a previous publication [27]. Briefly, to give context for the current investigation, this KBP model included various anatomical structures such as the Clinical Target Volume (CTV), Planning Target Volume (PTV), Carina, Esophagus, Great Vessels, Heart, Contralateral Lung, Ipsilateral Lung, Total Lung, Lungs-CTV, Spinal Cord, and Spinal Cord with a 5 mm margin. Additionally, the model incorporated 15 cardiac substructures. These substructures included the coronary arteries (left anterior descending, left circumflex, left main coronary, right coronary), the great vessels (ascending aorta, pulmonary artery, superior vena cava), the valves (atrial, mitral, pulmonary, and tricuspid), and the chambers of the heart (left atrium, right atrium, right ventricle, left ventricle). All anatomical structures described above (including the 15 cardiac substructures) were used during optimization. The model consisted of 28 patients with stage III NSCLC, all of whom received a treatment dose of 60 Gy in 30 fractions. A custom optimization template was generated for the cardiac-sparing RapidPlan model; these settings can be viewed in supplementary tables A1 and A2. A normal tissue objective with a priority of 125 was used to strategically penalize the dose gradient outside the target, aiming to reduce the dose to OARs. The objective parameters included distances to target, start dose, end dose, and fall-off values of 1 mm, 105%, 30%, and 15 mm^−1^, respectively.

### Field geometry

**Fig. 1:**
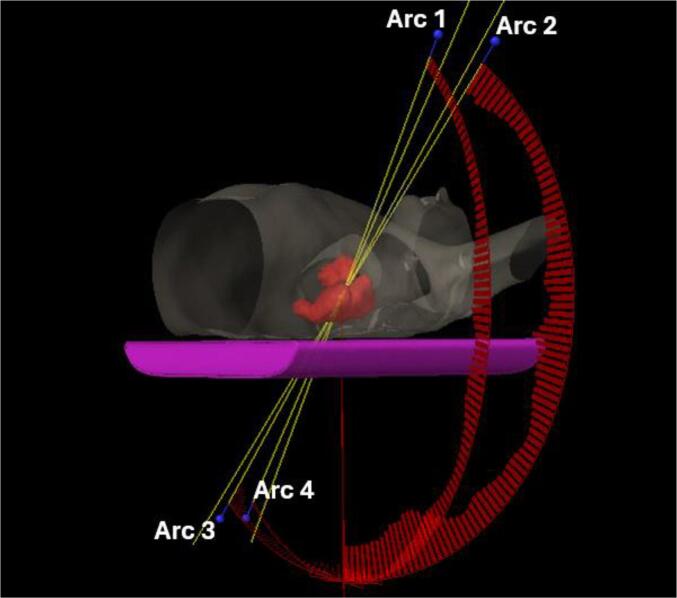
Example of field geometry for a representative case, with the PTV outlined in red. The beam arrangement consists of four noncoplanar partial arcs. Arc 1 – Gantry range: 55° CW 179.9°; Couch rotation: 10°. Arc 2 – Gantry range: 179.9° CCW 55°; Couch rotation: 350°. Arc 3 – 235° CCW 180.1°; Couch rotation: 5°. Arc 4 – 180.1° CW 235°; Couch rotation: 355°. (For interpretation of the references to colour in this figure legend, the reader is referred to the web version of this article.)

Two new treatment plans were generated using the cardiac-sparing model, each with different beam geometries. The first plan replicated the exact field geometry used in the clinically approved treatment plan but with the cardiac-sparing RapidPlan model applied (CS-Clinical). In contrast, the second plan used beam arrangements comprising noncoplanar oblique arc arrangements that preferentially avoid entry through the heart (CS-ncpOBL). These plans consist of 2–3 partial arcs entering through the ipsilateral lung. Additionally, 2–3 partial arcs crossing midline anteriorly or posteriorly (depending on the target location) were incorporated to spare healthy tissues. To further minimize the dose to healthy tissues, the couch was rotated to minimize entrance and exit doses through the heart, an example field arrangement can be seen in Fig. 1. Prior to optimization, clearance of all plans was verified using Radformation CollisionCheck software [14]. The Varian Eclipse Arc Geometry Tool (Version 16.1.0; Varian Palo Alto, CA) was employed to automatically adjust the field size to cover the PTV. If the field size in the X-direction exceeded 16 cm, the jaws were closed to 15–16 cm per the vendor's recommendations [28].

### Optimization

The cardiac-sparing model was applied to both plans, CS-Clinical and CS-ncpOBL. After selecting the model, optimization objectives were generated without any planner modifications. VMAT optimization was performed using the Varian Eclipse treatment planning system (Version 16.1.0), utilizing Photon Optimizer (version 16.1.1) and Acuros (version 16.1.0) for dose calculations. Inhomogeneity and air cavity corrections were on during optimization, and the convergence mode was enabled. Plans were normalized so that 100% of the prescription dose covered 95% of the PTV.

### Data analysis

The dose metrics used to calculate EDIC were recorded, including the mean dose (Gy) to the body, heart, and lungs. Body volume (cc) was also recorded. EDIC was calculated following the Jin model as described in Eq. (1) [23]. The mean and D0.03 cc were recorded for each of the 15 cardiac substructures. Statistical analysis was performed using the Wilcoxon signed-rank test to assess significant differences among the three planning groups (Clinical, CS-Clinical, CS-ncpOBL). The Bonferroni correction was applied with a resulting significance threshold of α = 0.01.

## Results

**Fig. 2:**
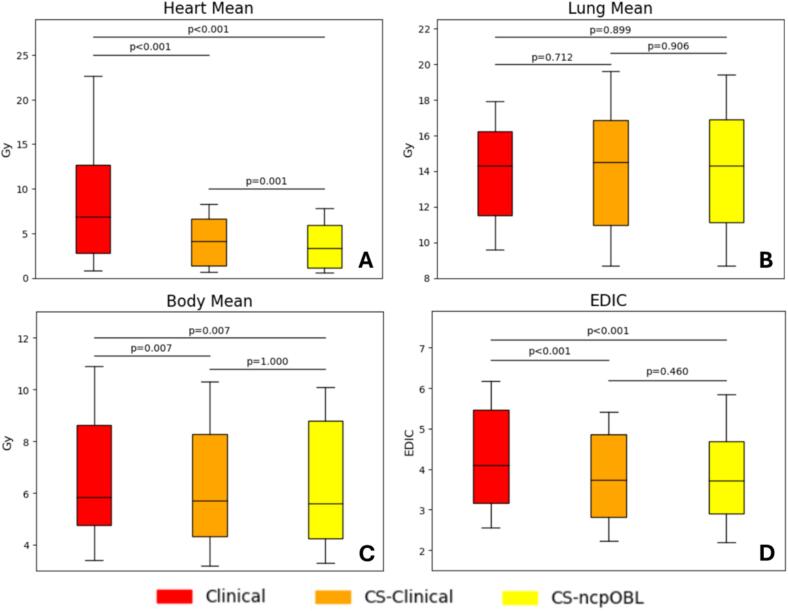
Box plots of the heart, lung, body and EDIC for 16 patients. Clinical = original, clinically treated plan; CS-Clinical = cardiac-sparing RapidPlan model with the clinical treatment plan field geometry; CS-ncpOBL = cardiac-sparing RapidPlan model with a patient-specific noncoplanar oblique field geometry.

The dosimetric endpoints analyzed for the 16 patients included in this study are detailed in Table 1 while Table 2 presents the corresponding p-values. Significant reductions in mean heart (p < 0.001) and mean body (p = 0.007) doses were observed in plans created using the cardiac-sparing RapidPlan model (CS-Clinical) compared to the Clinical plans. Specifically, mean heart doses decreased from 8.50 Gy to 4.09 Gy, and mean body dose decreased from 6.55 Gy to 6.18 Gy. Additionally, EDIC decreased from 4.27 to 3.81 (p < 0.001). When patient-specific treatment fields were utilized and planned with the cardiac-sparing RapidPlan model (CS-ncpOBL) compared to RapidPlan using the clinical field geometry (CS-Clinical), a statistically significant reduction in mean heart dose was observed (p = 0.001). The mean heart dose decreased from 4.09 Gy to 3.70 Gy, although there was no change in EDIC. Box plots in Fig. 2 depict the heart mean, lung mean, body mean, and EDIC for the three planning groups.

**Table 1:** Heart mean, lung mean, body mean, and EDIC averages were analyzed for 16 patients. Clinical = original, clinically treated plan; CS-Clinical = cardiac-sparing RapidPlan model with the clinical treatment plan field geometry; CS-ncpOBL = cardiac-sparing RapidPlan model with a patient-specific noncoplanar oblique field geometry.

**Plan**	**Heart Mean (Gy)**	**Lung Mean (Gy)**	**Body Mean (Gy)**	**EDIC**
Clinical	8.50 ± 6.91	13.92 ± 2.75	6.55 ± 2.39	4.27 ± 1.27
CS-Clinical	4.09 ± 2.73	13.97 ± 3.46	6.18 ± 2.39	3.81 ± 1.10
CS-ncpOBL	3.70 ± 2.50	14.00 ± 3.50	6.19 ± 2.40	3.79 ± 1.10

**Table 2:** Results of the Wilcoxon signed-rank test (threshold = 0.01) for EDIC, heart mean, lung mean, and body mean. Significant results are indicated by an asterisk (*). Clinical = original, clinically treated plan; CS-Clinical = cardiac-sparing RapidPlan model with the clinical treatment plan field geometry; CS-ncpOBL = cardiac-sparing RapidPlan model with a patient-specific noncoplanar oblique field geometry.

	**Clinical/CS-Clinical**	**Clinical/CS-ncpOBL**	**CS-Clinical/CS-ncpOBL**
Heart Mean	<0.001*	<0.001*	0.001*
Lung Mean	0.712	0.899	0.906
Body Mean	0.007*	0.007*	1
EDIC	<0.001*	<0.001*	0.460

Table 3 presents the mean dose and D0.03 cc values for all 15 cardiac substructures. Both CS-Clinical and CS-ncpOBL plans achieved significant reductions in mean dose and D0.03 cc compared to the Clinical plan across each cardiac substructure (p < 0.001). Notably, the CS-ncpOBL plans demonstrated further reductions in mean dose for the left anterior descending artery, left main coronary artery, and pulmonary valve relative to the CS-Clinical plans (p < 0.01). Both the CS-Clinical and CS-ncpOBL plans showed significant D0.03 cc reductions for the left anterior descending artery, left circumflex artery, left main coronary artery, right coronary artery, right atrium, right ventricle, aortic valve, mitral valve, pulmonary valve, and tricuspid valve compared to the Clinical plan (p < 0.01). The left ventricle sparing was significantly reduced for the CS-ncpOBL plans compared to the Clinical plans. Further dose sparing was observed in the CS-ncpOBL plans over the CS-Clinical plans specifically for the left anterior descending artery, left circumflex artery, left main coronary artery, and right ventricle (p < 0.01). Exact p-values for the mean and D0.03 cc doses across cardiac substructures are provided in supplementary tables A3 and A4.

**Table 3:** Comparison of mean dose and D0.03 cc across cardiac substructures for different treatment planning groups. Clinical = original, clinically treated plan; CS-Clinical = cardiac-sparing RapidPlan model with the clinical treatment plan field geometry; CS-ncpOBL = cardiac-sparing RapidPlan model with a patient-specific noncoplanar oblique field geometry.

**Substructure**	**Plan**	**Mean Dose (Gy)**	**D0.03 cc (Gy)**
Left Anterior Descending Artery	Clinical	16.78 ± 15.30	28.74 ± 21.84
	CS-Clinical	8.51 ± 10.12*	19.05 ± 21.83*
	CS-ncpOBL	7.56 ± 9.73†♦	17.62 ± 21.50†♦

Left Circumflex Artery	Clinical	21.71 ± 20.31	26.94 ± 23.56
	CS-Clinical	12.91 ± 15.31*	22.10 ± 24.72*
	CS-ncpOBL	11.64 ± 14.45†	20.40 ± 24.00†♦

Left Main Coronary Artery	Clinical	27.36 ± 18.91	32.19 ± 20.99
	CS-Clinical	11.14 ± 10.64*	16.71 ± 17.11*
	CS-ncpOBL	10.12 ± 11.18†♦	15.26 ± 17.66†♦

Right Coronary Artery	Clinical	9.79 ± 9.12	17.47 ± 14.86
	CS-Clinical	2.87 ± 2.31*	5.71 ± 5.92*
	CS-ncpOBL	2.71 ± 2.58†	4.74 ± 5.24†

Left Atrium	Clinical	16.31 ± 13.22	44.22 ± 23.51
	CS-Clinical	10.48 ± 8.69*	39.84 ± 27.81
	CS-ncpOBL	10.14 ± 8.80†	39.35 ± 28.06

Left Ventricle	Clinical	6.33 ± 7.08	29.40 ± 23.60
	CS-Clinical	2.68 ± 2.30*	21.68 ± 22.30
	CS-ncpOBL	2.35 ± 1.81†	20.53 ± 22.99†

Right Aorta	Clinical	6.38 ± 5.26	25.25 ± 18.22
	CS-Clinical	3.51 ± 3.44*	16.37 ± 17.18*
	CS-ncpOBL	3.16 ± 2.75†	15.72 ± 17.56†

Right Ventricle	Clinical	5.56 ± 4.83	23.62 ± 16.35
	CS-Clinical	2.04 ± 1.58*	11.15 ± 10.52*
	CS-ncpOBL	1.73 ± 1.43†	9.13 ± 10.41†♦

Ascending Aorta	Clinical	26.69 ± 11.77	54.02 ± 13.38
	CS-Clinical	13.08 ± 6.86*	48.23 ± 19.16
	CS-ncpOBL	12.71 ± 7.09†	47.46 ± 19.71

Pulmonary Artery	Clinical	35.32 ± 11.11	65.02 ± 1.73
	CS-Clinical	22.45 ± 7.53*	66.24 ± 1.35
	CS-ncpOBL	22.23 ± 7.73†	66.13 ± 1.76

Superior Vena Cava	Clinical	37.28 ± 17.14	54.04 ± 18.41
	CS-Clinical	26.46 ± 14.81*	51.62 ± 20.84
	CS-ncpOBL	27.11 ± 15.68†	51.26 ± 20.77

Atrial Valve	Clinical	14.63 ± 11.94	22.01 ± 17.47
	CS-Clinical	3.64 ± 2.68*	6.34 ± 5.19*
	CS-ncpOBL	3.52 ± 3.18†	6.43 ± 6.64†

Mitral Valve	Clinical	7.91 ± 8.15	13.69 ± 13.42
	CS-Clinical	2.73 ± 2.02*	4.60 ± 4.47*
	CS-ncpOBL	2.56 ± 1.96†	4.21 ± 4.33†

Pulmonary Valve	Clinical	20.92 ± 15.54	28.64 ± 20.24
	CS-Clinical	5.15 ± 4.71*	13.57 ± 17.47*
	CS-ncpOBL	4.55 ± 4.41†♦	13.24 ± 17.56†

Tricuspid Valve	Clinical	5.34 ± 5.66	6.71 ± 6.98
	CS-Clinical	1.68 ± 1.35*	2.45 ± 2.21*
	CS-ncpOBL	1.49 ± 0.92†	2.05 ± 1.43†

* p < 0.01 Clinical/CS-Clinical.

† p < 0.01 Clinical/CS-ncpOBL.

♦ p < 0.01 CS-Clinical/CS-ncpOBL.

**Fig. 3:**
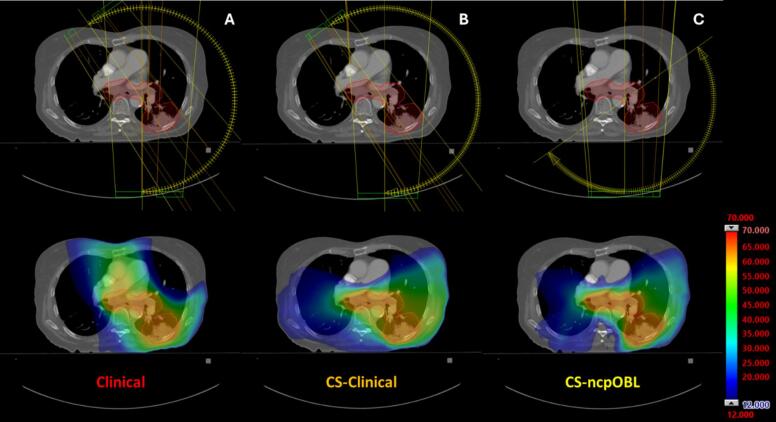
Field geometry and dose distribution comparison for a representative case. (A) Clinical = original, clinically treated plan; (B) CS-Clinical = cardiac-sparing RapidPlan model with the clinical treatment plan field geometry; (C) CS-ncpOBL = cardiac-sparing RapidPlan model with a patient-specific noncoplanar oblique field geometry.

Fig. 3 illustrates a comparison of field geometry and dose distribution across the three treatment planning methods in a representative case. The CS-Clinical plan achieved reductions in cardiac substructure and overall heart dose compared to the Clinical plan. Notably, the patient-specific noncoplanar oblique (CS-ncpOBL) plan provided the most decrease in cardiac substructure and mean heart doses. Although the CS-ncpOBL approach introduced some low-dose exposure to other organs-at-risk (OARs), there were no statistically significant differences in any of the other recorded metrics across the plans. All plans satisfied the clinical objectives outlined in RTOG 0617.

## Discussion

The importance of reducing dose to the heart as well as the impact of minimizing EDIC have been emphasized previously in the literature [29]. Prior work has demonstrated EDIC to not only be prognostic of overall survival in locally advanced NSCLC [25], but also predictive of severe lymphopenia, pneumonitis, and hospitalization. It is known that lymphopenia is associated with worse prognosis of cancer patients receiving immunotherapy [30] and prior work has confirmed EDIC’s prognostic value in the era of durvalumab [24]. With these data in mind, it is clear that minimizing EDIC and lymphopenia is of the utmost importance in the modern era of lung cancer treatment. For thoracic cancers, protocols typically target a mean heart dose below 20 Gy to reduce the risk of cardiac-related events [31], as elevated doses for cardiac structures have been linked to reduced overall survival rates [26]. The findings in this study presented that employing a cardiac-sparing RapidPlan model led to a significant reduction in heart dose and lowered EDIC without compromising overall plan quality, showing an average reduction in heart dose from 8.50 Gy to 4.09 Gy compared to the original clinical plans (p < 0.01). Cardiac substructure mean dose decreased for the plans using the original beam geometry with a cardiac-sparing RapidPlan model applied (CS-Clinical) compared to the original clinical plan (Clinical) (p < 0.001). The D0.03 cc cardiac substructure dose also decreased for 10/15 cardiac substructures (p < 0.01), indicating that tailoring specific models for distinct clinical scenarios may yield better outcomes compared to more generalized models, emphasizing the importance of targeted planning for improved clinical results [27].

When noncoplanar oblique beam geometry was employed in place of the standard field geometry, the average heart dose decreased from 4.09 Gy to 3.70 Gy, demonstrating the potential for enhanced dose reductions through strategic beam selection that avoided heart entry. The CS-ncpOBL approach achieved significantly lower mean doses to the left anterior descending artery, left main coronary artery, and pulmonary valve (p < 0.01), as well as D0.03 cc doses for the left anterior descending artery, left circumflex artery, left main coronary artery, and right ventricle (p < 0.01). The configuration of beam geometry plays a critical role in developing high-quality treatment plans, as supported by numerous studies [12], [13], [14], underscoring the importance of carefully designed beam arrangements in reducing cardiac dose. In this study, the CS-Clinical plan used the same field geometry as the Clinical plan, with 2–4 coplanar partial arcs over the ipsilateral lung to minimize dose to contralateral lung dose. However, due to the varied size and location of stage III lung tumors included, the standard clinical field arrangement proved suboptimal for cardiac sparing, prompting exploration of noncoplanar oblique arcs (illustrated in Fig. 3C). This alternative approach led to a substantial decrease in mean heart dose compared to the CS-Clinical plan (p = 0.001). The effectiveness of oblique arcs can be attributed to selecting beam angles along an arc trajectory that do not enter through the heart, positioning the heart at a deep depth dose on a tissue maximum ratio (TMR) table where the dose is lower. This decreases the integral body dose and subsequently reduces the effective dose to critical organs, as the mean body dose is a contributing factor in EDIC. By avoiding entry through the heart to reach the PTV, oblique arcs along with the strategic rotation of the treatment couch further minimized the entrance and exit dose to the heart. Although CS-ncpOBL plans did not yield a significant reduction in EDIC compared to CS-Clinical plans, a statistically significant decrease in heart dose and 4/15 cardiac substructures was achieved, demonstrating the cardiac-sparing advantage of noncoplanar oblique arcs in treatment planning.

The results from this study show that despite differences in field geometry, there was no significant differences in lung and body mean dose (Table 2 and Fig. 2B/2C). The comparison of KBP models with manual planning and the use of noncoplanar arcs to reduce cardiac dose have been previously explored in literature [12], [13], [27], [17], [18], [19]. However, existing KBP studies often lack detailed descriptions of beam geometry beyond that used in developing the KBP model. Consequently, there has been limited guidance on the optimal beam geometry for KBP models. This research highlights the importance of beam geometry when using KBP models. Given that plan quality improved with an advantageous beam geometry, it may be possible to retrain the KBP using other beam geometries for even better OAR sparing, though this was beyond the scope of the current project. Compared to manual planning, the cardiac-sparing KBP model demonstrated superior cardiac-sparing and reduction of EDIC and incorporating noncoplanar oblique arcs further reduced cardiac dose.

Treatment planning goals can change over time due to updates in the literature and shifts in clinical practice. The plans used as benchmarks in this study were treated during an era when high importance was placed on minimizing lung V5Gy as compared to minimizing heart dose. A shift in our practice has occurred since then, in part based on a secondary analysis of RTOG 0617 which demonstrated that lung V5Gy does not serve as a reliable predictor for radiation pneumonitis in NSCLC patients [32]. Moreover, lung V5Gy is not listed as a dosimetric constraint in the most recent NCCN lung guidelines [32], [33]. The differences in the low-dose wash in the lungs as observed in the Clinical plans (Fig. 3A) compared to the CS-Clinical plans (Fig. 3B) can be attributed to the recent shift in our clinical practice. In other clinics where lung V5Gy may be prioritized, the benchmark plans would likely differ as would relative gains in cardiac sparing. Nevertheless, this study raises awareness about shifting clinical goals and the importance of realigning optimization settings in light of new clinical data.

To treat arcs that cross midline posteriorly, the gantry must be rotated anteriorly around the patient. This anterior rotation, combined with noncoplanar beam arrangements, increases the risk of collision with the treatment table, patient, or support devices. This risk can be mitigated through strategic isocenter positioning and the use of collision-check software. Additionally, the gantry's limitation of not being able to rotate more than 5° past 180° in either direction can contribute to increased treatment times. Treatment delivery times were not recorded as a part of this study. While cardiac-sparing KBP models enhance treatment quality, contouring cardiac substructures can be time-consuming and may not be practical in all cases. Advancements in automated contouring software offer promising avenues to streamline and automate these processes, making the inclusion of intricate anatomical structures more feasible and realistic in the future. Future research should investigate other anatomical regions and assess the impact of substructure KBP models on plan quality.

## Conclusion

This study investigated radiation doses to the heart and immune cells for VMAT lung radiotherapy using a cardiac-sparing KBP model with coplanar and noncoplanar oblique beam geometries. As compared to clinically treated plans, the KBP model was shown to reduce heart mean dose, EDIC, and cardiac substructure dose. While noncoplanar oblique beams did not significantly reduce EDIC as compared to coplanar beams, heart dose was significantly lower without an increase in mean lung dose. These findings highlight the critical role of beam geometry in treatment planning when applying KBP models for VMAT lung radiotherapy.

## Declaration of competing interest

The authors declare that they have no known competing financial interests or personal relationships that could have appeared to influence the work reported in this paper.
