# Supplementary material for: Integrating knowledge-based planning and noncoplanar oblique VMAT arcs: A study of dose to the heart and immune cells in thoracic radiotherapy
Source: Tech Innov Patient Support Radiat Oncol. 2025 Jan 5;33:100301. doi: 10.1016/j.tipsro.2025.100301 (PMC11780144; doi:10.1016/j.tipsro.2025.100301)
Supplement: Supplementary Data 1 [file mmc1.docx]

**SUPPLEMENTARY MATERIAL**

Table A1: Cardiac-sparing RapidPlan model target objectives

| ID | Volume [%] | Dose [%] | Priority |
| --- | --- | --- | --- |
| **CTV** | | | |
| Upper | 0 | 105 | 125 |
| Upper | 1 | 104 | 125 |
| Upper | 5 | 103 | 125 |
| Lower | 100 | 100.5 | 125 |
| Lower | 99 | 101.5 | 125 |
| Lower | 98 | 102.5 | 125 |
| **PTV** | | | |
| Upper | 0 | 105 | 150 |
| Upper | 1 | 104 | 150 |
| Upper | 5 | 103 | 150 |
| Lower | 100 | 100 | 150 |
| Lower | 99 | 101 | 150 |
| Lower | 98 | 102 | 150 |

Table A2: Cardiac-sparring RapidPlan model organ-at-risk objectives

| ID | Volume [%] | Dose [Gy] | Priority |
| --- | --- | --- | --- |
| **Left anterior descending artery** | | | |
| Upper | Generated | 15 | Generated |
| Upper | Generated | 45 | Generated |
| Line (preferring target) | Generated | Generated | Generated |
| **Left circumflex artery** | | | |
| Line (preferring target) | Generated | Generated | Generated |
| **Left main coronary artery** | | | |
| Upper | Generated | 45 | Generated |
| Line (preferring target) | Generated | Generated | Generated |
| **Right coronary artery** | | | |
| Upper | 0 | 19.5 | Generated |
| Upper | Generated | 45 | Generated |
| Line (preferring target) | Generated | Generated | Generated |
| **Carina** | | | |
| Line | Generated | Generated | Generated |
| **Left atrium** | | | |
| Upper | Generated | 45 | Generated |
| Upper | Generated | 45 | Generated |
| Line (preferring target) | Generated | Generated | Generated |
| **Left ventricle** | | | |
| Upper | Generated | 45 | Generated |
| Line | Generated | Generated | Generated |
| **Right atrium** | | | |
| Upper |  |  |  |
| Line (preferring target) | Generated | Generated | Generated |
| **Right ventricle** | | | |
| Upper | Generated | 45 | Generated |
| Line (preferring target) | Generated | Generated | Generated |
| **Esophagus** | | | |
| Line (preferring target) | Generated | Generated | Generated |
| **Great vessels** | | | |
| Line (preferring target) | Generated | Generated | Generated |
| **Aorta** | | | |
| Upper | Generated | 45 | Generated |
| Mean |  | 8.5 | Generated |
| Line (preferring target) | Generated | Generated | Generated |
| **Pulmonary artery** | | | |
| Upper | Generated | 45 | Generated |
| Mean |  | 8.5 | Generated |
| Line (preferring target) | Generate | Generated | Generated |
| **Superior vena cava** | | | |
| Upper | Generate | 45 | Generated |
| Mean |  | 8.5 | Generated |
| Line (preferring target) | Generated | Generated | Generated |
| **Heart** |  |  |  |
| Line (preferring target) | Generated | Generated | Generated |
| **Contralateral lung** | | | |
| Line (preferring target) | Generated | Generated | Generated |
| **Ipsilateral lung** | | | |
| Line (preferring target) | Generated | Generated | Generated |
| **Lungs** |  |  |  |
| Line (preferring target) | Generated | Generated | Generated |
| **Lungs-CTV** |  |  |  |
| Line (preferring target) | Generated | Generated | Generated |
| **Spinal cord** |  |  |  |
| Line (preferring target) | Generated | Generated | Generated |
| **Spinal cord + 5 mm** | | | |
| Line (preferring target) | Generated | Generated | Generated |
| **Atrial valve** | | | |
| Line (preferring target) | Generated | Generated | Generated |
| **Mitral valve** | | | |
| Line (preferring target) | Generated | Generated | Generated |
| **Pulmonary valve** | | | |
| Line (preferring target) | Generated | Generated | Generated |
| **Tricuspid valve** | | | |
| Line (preferring target) | Generated | Generated | Generated |

Table A3: P-values for cardiac substructure mean (p<0.01)

|  | **Clinical/CS-Clinical** | **Clinical/CS-ncpOBL** | **CS-Clinical/CS-ncpOBL** |
| --- | --- | --- | --- |
| Left Anterior Decending Artery | <0.001* | <0.001* | 0.003* |
| Left Circumflex Artery | <0.001* | <0.001* | 0.044 |
| Left Main Coronary Artery | <0.001* | <0.001* | 0.006* |
| Right Coronary Artery | <0.001* | <0.001* | 0.323 |
| Left Atrium | <0.001* | <0.001* | 0.193 |
| Left Ventricle | <0.001* | <0.001* | 0.159 |
| Right Aorta | <0.001* | <0.001* | 0.298 |
| Right Ventricle | <0.001* | <0.001* | 0.144 |
| Ascending Aorta | <0.001* | <0.001* | 0.083 |
| Pulmonary Artery | <0.001* | <0.001* | 0.528 |
| Superior Vena Cava | <0.001* | <0.001* | 0.323 |
| Atrial Valve | <0.001* | <0.001* | 0.175 |
| Mitral Valve | <0.001* | <0.001* | 0.083 |
| Pulmonary Valve | <0.001* | <0.001* | 0.003* |
| Tricuspid Valve | <0.001* | <0.001* | 0.404 |

*Denotes a significant result

Table A4: P-values for cardiac substructure D0.03cc (p<0.01)

|  | **Clinical/CS-Clinical** | **Clinical/CS-ncpOBL** | **CS-Clinical/CS-ncpOBL** |
| --- | --- | --- | --- |
| Left Anterior Descending Artery | 0.002* | <0.001 | 0.008* |
| Left Circumflex Artery | 0.007* | 0.002* | 0.006* |
| Left Main Coronary Artery | <0.001* | <0.001* | 0.002* |
| Right Coronary Artery | <0.001* | <0.001* | 0.044 |
| Left Atruim | 0.231 | 0.159 | 0.900 |
| Left Ventricle | 0.013 | 0.005* | 0.495 |
| Right Aorta | <0.001* | <0.001* | 0.433 |
| Right Ventricle | <0.001* | <0.001* | 0.005* |
| Ascending Aorta | 0.083 | 0.011 | 0.083 |
| Pulmonary Artery | 0.029 | 0.044 | 0.980 |
| Superior Vena Cava | 0.111 | 0.074 | 0.940 |
| Atrial Valve | <0.001* | <0.001* | 0.821 |
| Mitral Valve | <0.001* | <0.001* | 0.083 |
| Pulmonary Valve | <0.001* | <0.001* | 0.252 |
| Tricuspid Valve | <0.001* | <0.001* | 0.231 |

*Denotes a significant result
